# Wet Aerobic Oxidation of Lignin into Aromatic Aldehydes Catalysed by a Perovskite-type Oxide: LaFe_1-x_Cu_x_O_3_ (x=0, 0.1, 0.2)

**DOI:** 10.3390/molecules14082747

**Published:** 2009-07-27

**Authors:** Junhua Zhang, Haibo Deng, Lu Lin

**Affiliations:** 1 Department of Resources Science and Engineering, State Key Laboratory of Pulp and Paper Engineering, South China University of Technology, Guangzhou 510640, Guangdong Province, China; 2 College of Textile and Clothing, Jiangnan University, Wuxi 214122, Jiangsu Province, China

**Keywords:** perovskite-type oxide, LaFe_1-x_Cu_x_O_3_, heterogeneous, wet aerobic oxidation, lignin, aromatic aldehydes

## Abstract

The perovskite-type oxide catalyst LaFe_1-x_Cu_x_O_3_ (x=0, 0.1, 0.2) was prepared by the sol–gel method, and tested as a catalyst in the wet aerobic oxidation (WAO) of lignin into aromatic aldehydes. The lignin conversion and the yield of each aromatic aldehyde were significantly enhanced in the catalytic process, compared with the non-catalyzed process. Moreover, it was shown that the stability of activity and structure of LaFe_1-x_Cu_x_O_3_ (x=0, 0.1, 0.2) remained nearly unchanged after a series of successive recyclings of the catalytic reactions, indicating it was an efficient and recyclable heterogeneous catalyst for the conversion of lignin into aromatic aldehydes in the WAO process.

## 1. Introduction

**Figure 1:**
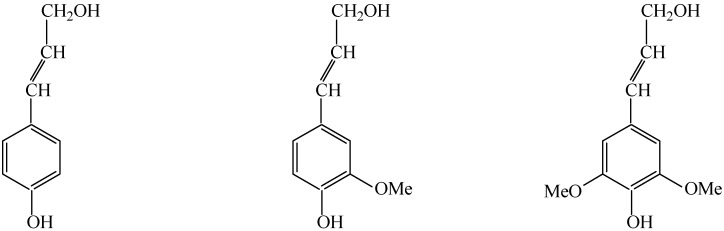
Lignin building units.

**Figure 2:**
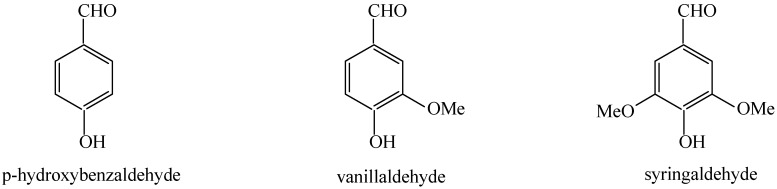
Aromatic aldehydes resulting from the lignin WAO process.

With the gradual diminishing of fossil fuel reserves and growing concerns about global warming, finding ways of exploiting feasible pathways for the replacement of the petroleum-based chemicals is highly desirable [1]. To meet the growing demand for this, biomass can serve as a sustainable source of renewable fuels and chemicals. It was estimated that a sustainable production of 1.3 billion dry tonnes of biomass per year can be achieved without significant changes in agricultural practices and food supplies, as reported by the U.S. Department of Agriculture (USDA) and U.S. Department of Energy (DOE) recently [2,3]. However, lignin, one of the main components of biomass in the bio-refining process, is usually discarded as non-cellulosic waste. Lignin is an extremely complex three-dimensional macromolecule with irregular structure, which results from random dehydrogenative polymerization of phenylpropane building units, including coniferylic, sinapylic and *р*-coumarylic alcohols (Figure 1), in the presence of peroxidase enzymes [4], and it can be converted into aromatic aldehydes such as *p*-hydroxybenzaldehyde, vanillaldehyde and syringaldehyde (Figure 2). These aromatic aldehydes have wide applications in flavoring, as chemical intermediates for pharmaceutical drugs and agricultural pesticides [5,6]. So the study of the conversion of aromatic aldehydes from lignin is of significant importance.

A routine way to convert lignin into aromatic aldehydes is Wet Aerobic Oxidation (WAO) process, which often uses oxygen and catalysts to increase the yields of aldehydes. Some noble metals have been used as catalysts in the lignin WAO process [6,7,8], but they are expensive, which is an impediment for their commercial applications. Alternatively, some inexpensive metal ions such as iron, copper, and cobalt have also shown activity in the lignin oxidation process [9,10,11,12,13]. Nevertheless, these homogeneous catalysts can lead to secondary pollution, result in high recycling costs, and restricting their industrial utilization.

It is well known that perovskite-type oxides of general formula ABO_3_ have high activity and stability in catalytic hydrocarbon oxidation processes, and they are a promising alternative to noble metal catalysts for the wet hydrocarbon catalytic oxidation process [14,15,16,17]. Nevertheless, the activity of perovskite-type oxides as catalysts in the WAO reaction has rarely been studied. To the best of our knowledge, only three recent studies deal with the activity of perovskite-type oxides La_1-x_A_x_BO_3_ (A=Sr, Ce; B=Co, Mn) for the WAO reaction [18,19,20], all claiming that this perovskite-type oxide presented high activity for this reaction. However, metal ions such as Sr, Ce, Co and Mn can cause a secondary pollution problem to a certain extent. For this reason, the WAO reaction of lignin is one process where the demand for green chemistry and sustainable technology is stimulating the replacement of these ‘‘toxic” metal ions by alternative “non-toxic” metal ions.

Iron (Fe) is the only transition metal which can be considered “non-toxic” to nature, so the study of LaFeO_3_ as a catalyst in the catalytic WAO of lignin is an inviting prospect. However, in the past decades, the effect of iron (Fe) on the hydrocarbon catalytic oxidation has rarely been studied due to its poor oxidation ability. In this paper, Cu ion was loaded into LaFeO_3_ to partially replace the Fe ion because the Cu ion has higher oxidation reaction activity [21,22,23], and a new catalyst: LaFe_1-x_Cu_x_O_3_ (x=0, 0.1, 0.2) was thus obtained. The activity and stability of this species as a catalyst were tested in the catalytic WAO of lignin into aromatic aldehydes, and LaFe_1-x_Cu_x_O_3_ (x=0, 0.1, 0.2) was also characterized by XRD, TPR and XPS.

## 2. Result and Discussion

### 2.1. Catalyst characterization

#### 2.1.1. Surface oxygen species and Fe ion oxidation states

**Figure 3:**
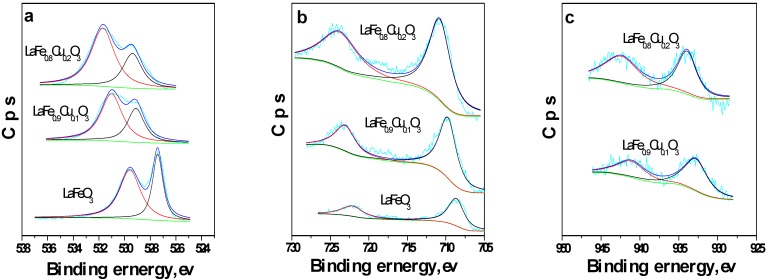
The XPS spectra of LaFe_1-x_Cu_x_O_3_ (x=0, 0.1, 0.2) (a. O 1s; b. Fe 2p; c. Cu 2p).

The O 1s, Fe 2p and Cu 2p XPS spectra of LaFe_1-x_Cu_x_O_3_ (x=0, 0.1, 0.2) are depicted in Figure 3. It can be seen from the O 1s spectrum (Figure 3a) that two contributions could be obtained by curve fitting of O 1s, which are assigned to lattice oxygen in the form of O^2-^ (metal oxygen bond) and adsorbed oxygen species (such as O^-^, O_2_^-^ or O_2_^2-^), respectively, according to the prior literature [24,25]. From the integral of O 1s spectrum, it can be found that the content of oxygen increased as the x value of LaFe_1-x_Cu_x_O_3_ increased (x=0, 0.1, 0.2), indicating that the absorption ability of oxygen increases as the content of copper ion increases, a result similar to that obtained by Zhang *et al*. [26]. Moreover, in Figure 3(b) the appearance of two signals can be observed, which belong to Fe 2p3/2 and Fe 2p1/2, respectively, indicating that there are Fe^3+^ ions in the catalyst, as indicated in [27]. Furthermore, two signals at BE=930-935 and BE=940-955 ev can be also observed from Figure 3(c), which belong to Cu^2+^ according to the literature [28]. All these indicated that the LaFe_1-x_Cu_x_O_3_ (x=0, 0.1, 0.2) catalyst was a typical perovskite-type oxide.

#### 2.1.2. TPR profile of LaFe_1-x_Cu_x_O_3_ (x=0, 0.1, 0.2)

**Figure 4:**
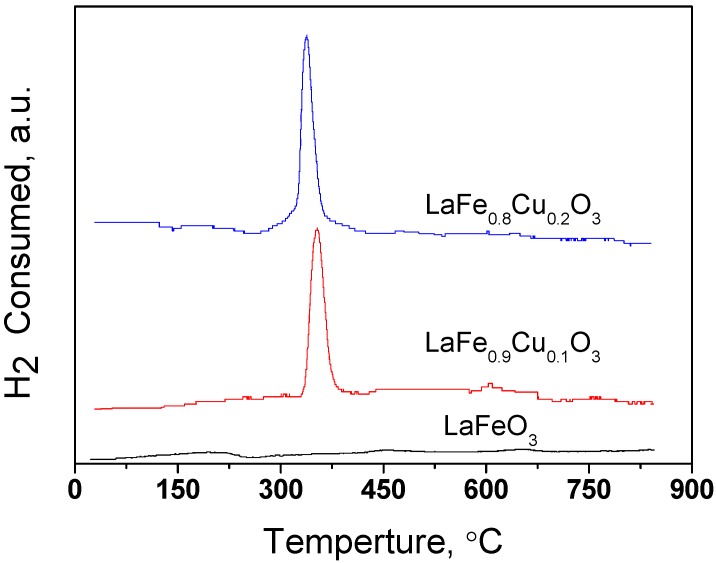
TPR profile of the catalysts LaFe_1-x_Cu_x_O_3_ (x=0, 0.1, 0.2).

The reducibility of LaFe_1-x_Cu_x_O_3_ (x=0, 0.1, 0.2) samples is depicted in Figure 4. An obvious LaFe_1-x_Cu_x_O_3_ (x=0, 0.1, 0.2) reducing peak can be observed as the x value decreases from 0.2 to 0, which is attributed to the reduction of Cu^2+^ according to the literature cited [29,30,31], indicating that the Cu has attached to the LaFe_1-x_Cu_x_O_3_ (x=0, 0.1, 0.2) catalyst as the x value increased.

### 2.2. Catalytic oxidation lignin into aromatic aldehydes

**Figure 5:**
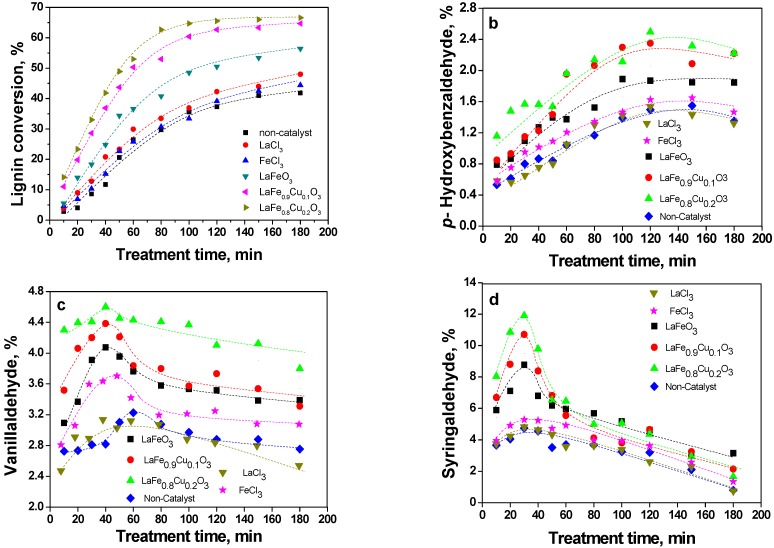
Lignin conversion (a), yield of *p*-hydroxybenzaldehyde (b), vanillaldehyde (c) and syringaldehyde (d) with reaction time (the reaction conditions were as follows: NaOH (2 mol/L), 120 °C, 5 bar partial pressure of oxygen in 20 bar total pressure, C_L0_=60.00 kg/m^3^, C_catalyst_=3 g/L and the lignin conversion=(C_0_-C_t_)/C_0_, where C_0_ is the initial concentration of lignin, and C_t_ is the concentration of lignin at any reaction time).

The aromatic aldehydes obtained from lignin in the catalyzed WAO process can be further oxidized to aromatic acids, and even to carbon dioxide and water under certain conditions. In our experiment, the catalytic activity was evaluated with the rate of lignin conversion and the yield of aldehydes, which was shown in Figure 5. It can be seen from Figure 5(a) that the lignin conversion was 41.8% at 3.0 hours in the absence of a catalyst, and the conversion gradually increased when LaFeO_3_, LaFe_0.9_Cu_0.1_O_3_ and LaFe_0.8_Cu_0.2_O_3_ were added, respectively. The maximum lignin conversion (66.6%) could be reached after 3.0 hours when lignin oxidation was catalysed by LaFe_0.8_Cu_0.2_O_3_, which was 1.59 times the yield obtained in the non-catalytic process. Similar concentrations of LaCl_3_ and FeCl_3_ did not present any catalytic activity under these conditions.

The yields of *p*-hydroxybenzaldehyde, vanillaldehyde and syringaldehyde are shown in Figures 5(b-d). The same results were obtained when LaCl_3_, FeCl_3_, LaFeO_3_, LaFe_0.9_Cu_0.1_O_3_ and LaFe_0.8_Cu_0.2_O_3_ were added, respectively, and the yields increased gradually. The maximum yields of *p*-hydroxy-benzaldehyde, vanillaldehyde and syringaldehyde were 2.49% (at 120 min), 4.56% (at 60 min) and 11.51% (at 30 min) in the LaFe_0.8_Cu_0.2_O_3_ catalytic process, which were 1.66, 1.42 and 2.51 times those obtained in the non-catalytic process, respectively. All results indicate that the catalyst activity will be enhanced by an increase in Cu content.

**Figure 6:**
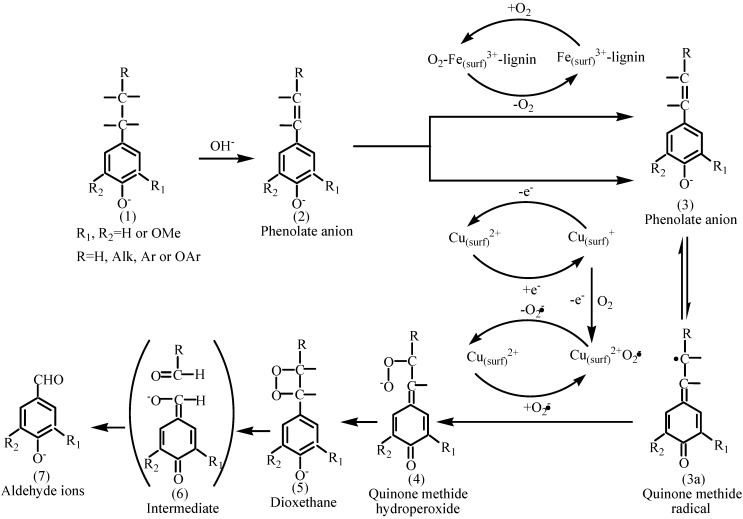
Proposed mechanism of oxidation of lignin in the LaFe_1-x_Cu_x_O_3_ (x=0, 0.1, 0.2) catalyst process.

Although it is premature to discuss the precise role of the perovskite oxide LaFe_1-x_Cu_x_O_3_ (x=0, 0.1, 0.2) in the catalytic mechanism of the lignin oxidation process at present, it is noteworthy that two aspects can account for the activity of LaFe_1-x_Cu_x_O_3_ (x=0, 0.1, 0.2) in the CWAO as reported [18]: On the one hand, an O_2_-Fe(surf)^3+^-lignin intermediate was generated on the surface of LaFe_1-x_Cu_x_O_3_ (x=0, 0.1, 0.2), which will enhance the oxygen contact of lignin and accelerate the generation of the intermediate of quinine methide radical: (3) or (3a); On the other hand, the oxygen space will be enhanced with the partial replacement of Fe^3+^ by Cu^2+^, according to the report of Zhang [17], which would accelerate the oxygen surface absorption ability of the catalyst and the intermediate content of O_2_-Fe(surf)^3+^-lignin will be enhanced. Moreover, the amount of activated species Cu_(surf)_^2+^ O_2_^.-^ will be increased with the partial replacement of Fe^3+^ by Cu^2+^, which will result a cycling of Cu_(surf)_^2+^→ Cu_(surf)_^+^ →Cu_(surf)_^2+^ O_2_^.-^→Cu_(surf)_^2+^ and accelerate the generation of the intermediate quinone methide radical: (3) or (3a) and quinone methide hydroperoxide : (4). With the combined effect of the above mentioned factors, the catalytic activity of LaFe_1-x_Cu_x_O_3_ (x=0, 0.1, 0.2) is improved. The proposed mechanism of oxidation of lignin catalysted by LaFe_1-x_Cu_x_O_3_ (x=0, 0.1, 0.2) was depicted in Figure 6.

**Figure 7:**
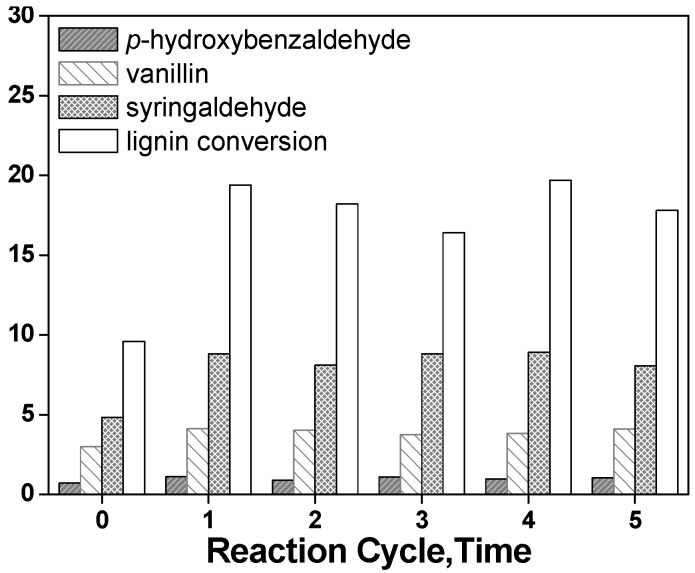
Lignin conversion and yield of aromatic aldehydes in the absence of catalyst (process 0) and five successive runs of LaFeO_3_ for 30 minutes (other reaction conditions as indicated in Figure 5).

The catalytic stability of LaFeO_3_ was investigated by repeatedly using LaFeO_3_ for the lignin conversion under the same reaction conditions; the lignin conversion and yield of aromatic aldehydes from the non-catalyzed process and five successive runs is shown in Figure 7, where it can be seen that the lignin conversion and the yield of each aromatic aldehyde were significantly higher in each of successive catalyst run than the non-catalyzed process. Furthermore, it was clearly demonstrated that the lignin conversion and the yield of aromatic aldehydes remained nearly the same in each run, which indicated that the catalyst has better recovery ability.

**Figure 8:**
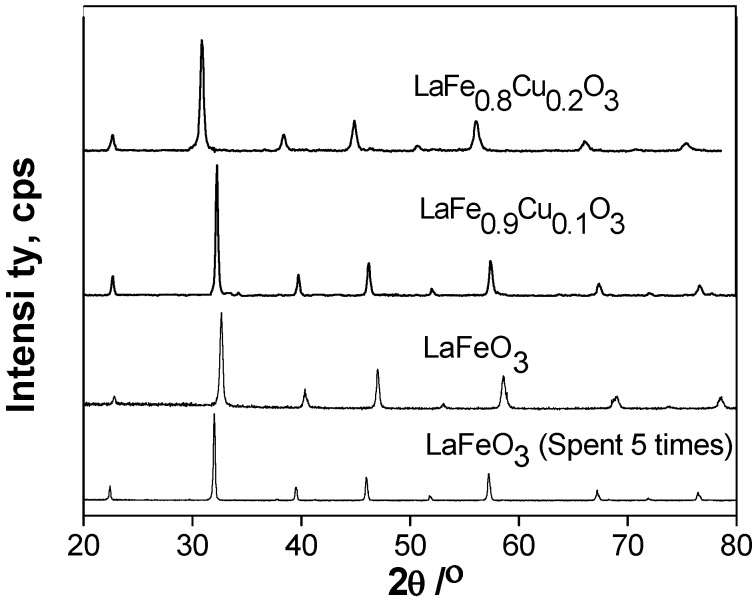
XRD patterns of LaFeO_3_, LaFe_0.9_Cu_0.1_O_3_, LaFe_0.8_Cu_0.2_O_3_ and LaFeO_3_ which was used five times.

The crystalline phases of the catalysts before and after use were also determined by X-ray diffraction, which is depicted in Figure 8. The XRD patterns of the LaFeO_3_, LaFe_0.9_Cu_0.1_O_3_, LaFe_0.8_Cu_0.2_O_3_ and LaFeO_3_ which were used five times all showed characteristic reflections for the perovskite-type oxide without other phases. No obvious changes can be seen in the structure of the LaFeO_3_ catalyst used five times, which suggested that the LaFeO_3_ catalyst is stable in the CWAO process.

## 3. Experimental

### 3.1. Lignin and catalyst preparation

Lignin was obtained through the process of enzymatic hydrolysis of steam-explosion cornstalks [32]. The LaFe_1-x_Cu_x_O_3_ (x=0, 0.1, 0.2) catalyst was prepared from citrate precursors [18,20,33]: a concentrated solution of metal nitrates was mixed with an aqueous solution of citric acid, and the molar ratio of citric acid to metal cations was fixed at 1.5. Water was evaporated from the solution at 80 °C until a viscous gel was obtained. The gel was kept at 100 °C overnight, and then calcined at 700 °C for 6 h.

### 3.2. Catalyst characterization *[20]*

X-ray diffraction (XRD) measurements were performed on a Rigaku powder diffractometer (Rigaku, Japan) with CuK_α_ radiation. The tube voltage was 45 kV, and the current was 40 mA. The XRD diffraction patterns were taken over 2 h in a range of 20° to 80° at a scan speed of 2°/min.

Temperature programmed reduction (TPR) of H_2_ was performed in a Micromeritics Autochem 2910 equipped with a thermal conductivity detector (TCD). The sample (50 mg) was pretreated in 10% O_2_/He flow at 800 °C for 30 min, and then it was reduced with 5 % H_2_/Ar (30 cm^3^ /min) heating 5 °C /min from room temperature to 850 °C.

The X-ray photoelectron spectroscopy (XPS) analysis was performed on a Kratos Axis Ultra system with 0.1 eV per step for detail scan and the binding energies for each spectrum were calibrated with a C1s spectrum of 284.6 eV. The core levels of O 1s, Fe 2p and Cu 2p species were recorded and their relative intensities determined.

### 3.3. Catalytic experiments *[20]*

The non-catalyzed WAO and catalyzed WAO processes of lignin were carried out in a high-pressure SS-316 Parr slurry reactor (model 4843) at 120 ± 1 °C. A 500 mL alkaline solution (NaOH, 2 mol/L) of lignin dissolved at a concentration of 60 g/L was introduced into the reactor and a certain quantity of catalysts was added to the solution if necessary. The heating program was started under a slight nitrogen pressure. When the solution in the reactor reached the desired temperature, nitrogen was added until a total pressure of 15 bars was attained, and then, the oxygen was added until the total pressure attained 20 bars. The pressure in the reactor was kept at 20 bars by continuous flushing of oxygen as a supplement because of its consumption during the reaction. Time was recorded from zero. During each reaction, sampling was conducted from the reactor and filtrated, and the filtration was acidified to pH 2-3 with a dilute HCl solution. The resulting products were extracted with chloroform until the chloroform layer appeared colorless. The residual lignin was obtained by centrifugation of the suspensions after the extraction of resulted products.

The contents of *p*-hydroxybenzaldehyde, vanillaldehyde and syringaldehyde in the chloroform extracts were analyzed by high performance liquid chromatography with a Hypersil ODS_2_ column (4.6 mm × 250 mm) and a UV detector set at 280 nm, and a mixture of acetonitrile (10%), deionized water (90%) and acetic acid (1.5%) as the mobile phase. The non-converted lignin was diluted in a sodium hydroxide solution to dissolve the lignin and a UV spectrophotometer used to determine the content of the non-converted lignin at a wavelength of 280 nm [34].

The experiment to test the recycling ability of LaFeO_3_ was performed as follows. Fresh catalyst and lignin solution were added into the reactor and the process was performed under the same conditions as mentioned above for 30 min. After the catalyzed WAO reaction, the reactor temperature was quickly cooled to room temperature, and the resulting products were carefully poured out and filtered. The catalyst was left in the reactor, and fresh lignin solution was added, then the process was performed again under the same conditions for 30 minutes. This procedure was repeated for four times, and the contents of *p*-hydroxybenzaldehyde, vanillaldehyde, syringaldehyde and lignin were analyzed using the above methods.

## 4. Conclusions

The perovskite-type oxide catalyst of LaFe_1-x_Cu_x_O_3_ (x=0, 0.1, 0.2) prepared by the sol–gel method exhibited high activity in the catalyzed WAO of lignin. Compared with the non-catalyzed process, the lignin conversion and yield of each aromatic aldehyde were improved significantly in the catalyzed process. It was shown that the perovskite-type oxide catalyst of LaFe_1-x_Cu_x_O_3_ (x=0, 0.1, 0.2) also possesses distinctive stability of activity and structure in the catalyzed WAO of lignin, and is thus an efficient and recyclable heterogeneous catalyst for the conversion of lignin in the catalyzed WAO process of lignin.
